# Global and Local Connectivity Differences Converge With Gene Expression in a Neurodevelopmental Disorder of Known Genetic Origin

**DOI:** 10.1093/cercor/bhx027

**Published:** 2017-07-01

**Authors:** Joe Bathelt, Jessica Barnes, F Lucy Raymond, Kate Baker, Duncan Astle

**Affiliations:** 1 MRC Cognition & Brain Sciences Unit, Cambridge CB2 7EF, UK; 2 Department of Medical Genetics, Cambridge Institute for Medical Research, University of Cambridge, Cambridge CB2 0XY, UK

**Keywords:** atypical brain development, cognitive development, human genetics, structural connectome

## Abstract

Knowledge of genetic cause in neurodevelopmental disorders can highlight molecular and cellular processes critical for typical development. Furthermore, the relative homogeneity of neurodevelopmental disorders of known genetic origin allows the researcher to establish the subsequent neurobiological processes that mediate cognitive and behavioral outcomes. The current study investigated white matter structural connectivity in a group of individuals with intellectual disability due to mutations in *ZDHHC9*. In addition to shared cause of cognitive impairment, these individuals have a shared cognitive profile, involving oromotor control difficulties and expressive language impairment. Analysis of structural network properties using graph theory measures showed global reductions in mean clustering coefficient and efficiency in the *ZDHHC9* group, with maximal differences in frontal and parietal areas. Regional variation in clustering coefficient across cortical regions in *ZDHHC9* mutation cases was significantly associated with known pattern of expression of *ZDHHC9* in the normal adult human brain. The results demonstrate that a mutation in a single gene impacts upon white matter organization across the whole-brain, but also shows regionally specific effects, according to variation in gene expression. Furthermore, these regionally specific patterns may link to specific developmental mechanisms, and correspond to specific cognitive deficits.

## Introduction

Many cognitive and psychiatric disorders are highly heritable (Haworth et al. 2009; Lee et al. 2015). In some cases, genetic risk factors have been identified, but understanding the neural mechanisms linking altered gene transcripts to cognitive or behavioral outcomes remains challenging. One reason for this is the heterogenous nature of the vast majority of these disorders, which presents a major challenge to establishing the neural endophenotypes that mediate any gene–cognition relationships; any group defined on the basis of a cognitive impairment or behavioral difficulty will likely contain individuals with different genetic and neural causes, making it difficult to identify mechanisms at the group level. One promising approach has been to study neuroanatomical differences in groups of individuals that have rare but clearly defined genetic causes of those impairments (Meyer-Lindenberg 2009; Griffa et al. 2013). These groups, while necessarily small in size, have a homogenous etiology. Studying these groups can therefore provide a powerful means for identifying the neurobiological pathways that potentially mediate cognitive and behavioral phenotypes in the wider population. For instance, the study of a rare familial speech disorder (KE family, FOXP2 mutation) highlighted the importance of striatal networks for emergent higher-order language skills (Liegeois et al. 2011; Watkins 2011).

However, studies of brain differences have mainly focussed on focal differences in brain areas or white matter tracts that show the most pronounced group differences. This is true of both genetically defined group comparisons and case-control designs more generally. However, genetic differences are likely to have wide-ranging effects on the organization of neural ensembles across many areas. To explore this fully requires a more advanced network science approach, capable of establishing how organizational principles differ across groups of individuals (Meyer-Lindenberg 2009; Petersen and Sporns 2015). We take this approach here.

In a network analysis, brain regions are described as nodes and their connections as edges. Nodes typically correspond to regions of interest (ROI) (Dell’Acqua and Catani 2012; Fornito et al. 2015). In the current study, edges represented fractional anisotropy (FA) of white matter connections between the regions. FA indicates anisotropic diffusion of water (Alexander et al. 2011). Higher levels of FA are associated with more ordered organization of axons and increased myelination (Feldman et al. 2010). Higher values of FA have been linked to higher cognitive performance (Clayden et al. 2011; Navas-Sanchez et al. 2013) and lower levels to white matter damage in patient studies (Kubicki et al. 2005; Verstraete et al. 2013). Organizational principles of the structural brain network can be quantified using graph theory (Bullmore and Sporns 2009; Rubinov and Sporns 2010). These approaches have been used to study typical and atypical brains across the lifespan (Hagmann et al. 2012; Collin and van den Heuvel 2013; Griffa et al. 2013; Deco and Kringelbach 2014; Martino et al. 2014).

A few studies have employed this network analysis approach to investigate how genetic differences may influence brain organization (Ottet et al. 2013; Hong et al. 2014; Leow et al. 2014; Meoded et al. 2014; Bruno et al. 2016). These studies focused on common variants of trophic factor genes (Meoded et al. 2014), genes involved the regulation of synaptic weights (Ottet et al. 2013; Hong et al. 2014; Meoded et al. 2014), and mutations associated with specific phenotypes (Leow et al. 2014; Bruno et al. 2016). Genetic differences were associated with differences in structural brain network organization, with specific effects for each genetic factor. This suggests that studying differences in brain organization may offer important insight into understanding the effects of genetic variation.

In the present study we take a network analysis approach to studying brain organization in a neurodevelopmental disorder defined by specific genetic origin. Mutations in *ZDHHC9* are a recurrent cause of X-linked Intellectual Disability (XLID) (Raymond et al. 2007). The *ZDHHC9* gene codes for a palmitoylation enzyme, involved in post-translational modification of specific target substrates. Palmitoylation plays an important role in the recruitment of receptors and ion channels at the synapse (Topinka and Bredt 1998; El-Husseini et al. 2000; Young et al. 2013). A systematic assessment of clinical history and cognitive deficits across multiple XLID-associated genes led to the observation that *ZDHHC9* mutations are associated with homogeneous neurological and cognitive features, including disproportionate attention problems, language impairment, and deficits in oromotor control in the context of mild to moderate intellectual disability (Baker et al. 2015). The majority of affected individuals also had a history of epilepsy that resembled Rolandic epilepsy in presentation and spike topography (Baker et al. 2015). Previous neuroimaging work in our group investigated focal differences in brain structure in *ZDHHC9* cases. These studies indicated differences in subcortical volumes (thalamus, putamen, and caudate nucleus) and hypoplasia of the corpus callosum (Baker et al. 2015). Reductions in cortical thickness were found that were most pronounced in areas around the temporoparietal junctions and inferior frontal lobe (Bathelt et al. 2016). Mutation of *ZDHHC9* was also associated with reductions in white matter structural integrity involving cortical, corticosubcortical, and interhemispheric tracts (Bathelt et al. 2016).

Given strong evidence for pervasive effects on white matter integrity, we expected the *ZDHHC9* mutation would have an impact on structural brain network organization. More specifically, we predicted that in addition to any global impact of gene mutation, we ought to observe some regional specificity in network properties, according to variability in the expression of that gene across the brain. This regional specificity may correspond to the areas of most marked cognitive impairment resulting from the mutation, and overlap with other genes known to result in similar phenotypic features, potentially via similar developmental mechanisms. In short, across our analyses we explored how both a mutation to, and regional expression of, *ZDHHC9* are associated with structural brain organization.

## Participants and Methods

### Participants

The study compared 7 males with inherited loss of function mutations in the *ZDHHC9* gene (age in years: mean = 29.13, standard error (SE) = 4.86, range = 13.83–41.83) to 7 males individually matched in age ± 2 years (age in years: mean = 27.23, SE = 5.31, range = 10.17–42.5). Comparison subjects had no history of neurological illness or cognitive impairment. Statistical analysis indicated no significant difference in age between the groups (Welch-corrected *t*-test: *t*(11.91) = −0.265, *P* = 0.796).

For detailed description of clinical and cognitive characteristics of the *ZDHHC9* group see Baker et al. 2015. In summary, all individuals with a *ZDHHC9* mutation had mild to moderate intellectual disability (full-scale IQ: mean = 64.86, SE = 2.32, range = 57–73). Five individuals had a history of epilepsy, with seizure characteristics and EEG features similar to the Rolandic epilepsy spectrum. At the time of magnetic resonance imaging (MRI) acquisition, 1 participant reported seizures within the previous 3 months, and 3 currently received antiepileptic medication (carbemazapine *n* = 1, carbemazapine and lamotrigine *n* = 1, phenytoin *n* = 1). Vineland scores (Sparrow et al. 2005) indicated stronger receptive language abilities compared with expressive and written language abilities in the *ZDHHC9* group. The Verbal Motor Production Assessment for Children (VMPAC) (Hayden and Square 1999) indicated significant oromotor difficulties in the *ZDHHC9* group, including deficits in oral control, sequencing, voice characteristics, and connected speech. Inhibitory control was also reduced in the *ZDHHC9* group on a visual attention task. These specific features differentiated with *ZDHHC9* group from age and IQ matched controls (Baker et al. 2015).

### MRI Data Acquisition

MRI data was acquired at the MRC Cognition and Brain Sciences Unit, Cambridge, UK. All scans were obtained on the Siemens 3 T Tim Trio system (Siemens Healthcare, Erlangen, Germany), using a 32-channel quadrature head coil. The imaging protocol consisted of 2 sequences: T1-weighted MRI and a diffusion-weighted sequence.

T1-weighted volume scans for surface analysis were acquired using a whole brain coverage 3D Magnetizable Prepared Rapid Acquisition Gradient Echo (MP RAGE) sequence acquired using 1 mm isometric image resolution. Echo time was 2.98 ms, and repetition time was 2250 ms. T1-weighted volumes were visually inspected for motion artefacts and were deemed sufficient by a radiographer not involved in the study.

Diffusion scans were acquired using echo-planar diffusion-weighted images with an isotropic set of 60 noncollinear directions, using a weighting factor of *b* = 1000 s mm^−2^, interleaved with 4 T2-weighted (*b* = 0) volumes. Whole brain coverage was obtained with 60 contiguous axial slices and isometric image resolution of 2 mm. Echo time was 90 ms and repetition time was 8400 ms.

Motion was quantified as the root-mean-square difference between volumes and the first volume in the diffusion sequence using the FMRIB Software Library (FSL)’s rmsdiff tool. The maximum displacement was under 3 mm for both *ZDHHC9* cases and controls (*ZDHHC9*: mean = 2.1, SE = 0.292, median = 1.97, mad = 0.648; control: mean = 1.8, SE = 0.304, median = 1.53, mad = 0.314) and there was no significant difference between groups (paired *t*-test: *t*(6) = −0.59, *P* = 0.58; Wilcoxon signed-rank test: *W* = 10, *P* = 0.499). There were also no differences in the number of outliers identified by FSL eddy (Andersson and Sotiropoulos 2016) (*ZDHHC9*: mean = 12.71, SE = 2.254, median = 11.0, mad = 5.93; control: mean = 10.0, SE = 3.078, median = 8.0, mad = 5.93; paired *t*-test: *t*(6) = −1.09, *P* = 0.317; Wilcoxon signed-rank test: *W* = 8, *P* = 0.311).

### Structural Connectome Analysis

**Figure 1:**
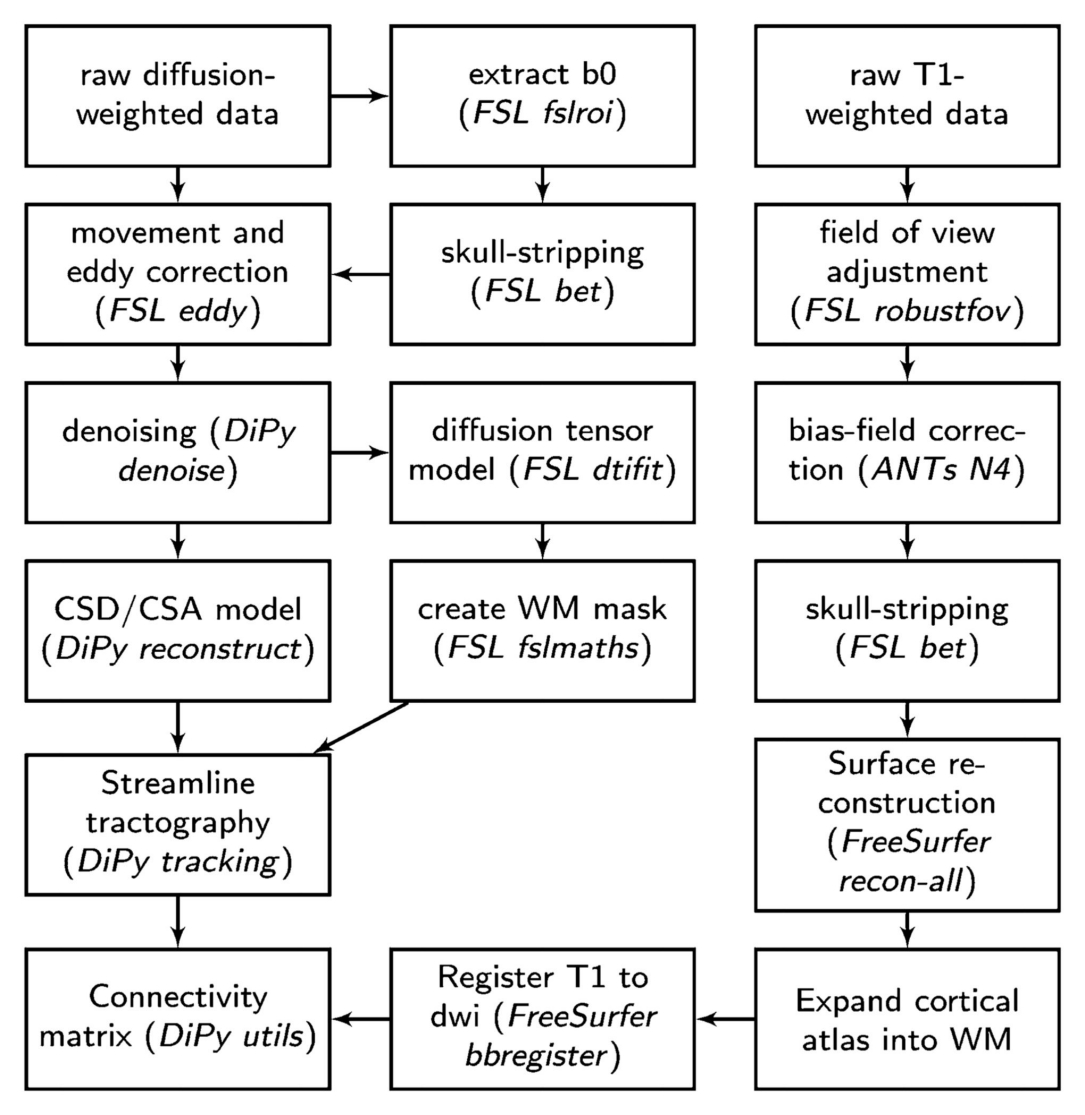
Overview of the processing steps to derive the diffusion-weighted structural connectome.

The white-matter connectome reconstruction followed the general procedure of estimating the most probably white matter connections for each individual, and then obtaining measures of FA between regions (Fig. 1). The details of the procedure are described in the following paragraphs.

In the current study, MRI scans were converted from the native DICOM to compressed NIfTI-1 format using the dcm2nii tool http://www.mccauslandcenter.sc.edu/mricro/mricron/dcm2nii.html. Subsequently, a brain mask was derived from the b0-weighted volume of the diffusion-weighted sequence and the entire sequence was submitted for correction for participant movement and eddy current distortions through FSL’s eddy tool. Next, nonlocal means denoising (Coupe et al. 2008) was applied using the Diffusion Imaging in Python (DiPy) v0.11 package (Garyfallidis et al. 2014) to boost signal to noise ratio. The diffusion tensor model was fitted to the preprocessed images to derive maps of FA using dtifit from the FMRIB Software Library (FSL) v.5.0.6 (Behrens et al. 2003). A spherical constrained deconvolution (CSD) model (Tournier et al. 2008) was fitted to the 60-gradient-direction diffusion-weighted images using a maximum harmonic order of 8 using DiPy. An alternative analysis with a constant solid angle (CSA) model is present in the Supplementary Materials section. Next, probablistic whole-brain tractography was performed based on the CSD model with 8 seeds in any voxel with a General FA value higher than 0.1. The step size was set to 0.5 and the maximum number of crossing fibers per voxel to 2.

For ROI definition, T1-weighted images were preprocessed by adjusting the field of view using FSL’s robustfov, nonlocal means denoising in DiPy, deriving a robust brain mask using the brain extraction algorithm of the Advanced Normalization Tools (ANTs) v1.9 (Avants et al. 2009), and submitting the images to recon-all pipeline in FreeSurfer v5.3 (http://surfer.nmr.mgh.harvard.edu). ROIs were based on the Desikan-Killiany parcellation of the MNI template (Desikan et al. 2006) with 34 cortical ROIs per hemisphere and 17 subcortical ROIs (brain stem, and bilateral cerebellum, thalamus, caudate, putamen, pallidum, hippocampus, amygdala, nucleus accumbens). The surface parcellation of the cortex was transformed to a volume using the aparc2aseg tool in FreeSurfer. Further, the cortical parcellation was expanded by 2 mm into the subcortical white matter using in-house software. In order to move the parcellation into diffusion space, a transformation based on the T1-weighted volume and the b0-weighted image of the diffusion sequence was calculated using FreeSurfer’s bbregister and applied to volume parcellation.

For each pairwise combination of ROIs, the number of streamlines intersecting both ROIs was estimated and transformed to a density map. A symmetric intersection was used, that is, streamlines starting and ending in each ROI were averaged. Spurious connections in streamline tractography are a common problem in structural connectome studies (Zalesky et al. 2016). Typically, a threshold is applied to remove false positive streamlines. However, the choice of this cut-off is largely arbitrary. In order to remove the effect of setting any particular threshold, a range of thresholds was applied and the area under the curve for each metric was compared in subsequent analyses (Wijk et al. 2010).

The weight of the connection matrices was based on FA. To obtain FA-weighted matrices, the streamline density maps were binarized after thresholding and multiplied with the FA map and averaged over voxels to obtain the FA value corresponding to the connection between the ROIs. This procedure was implemented in-house based on DiPy v0.11 functions (Garyfallidis et al. 2014). Edge weights may be defined in different ways (Qi et al. 2015), which may considerably influence the results of the analysis (Fornito et al. 2013). Therefore, additional analyses were carried out with alternative edge weight definitions, that is, streamline count, streamline count normalized by ROI size, and streamline count normalized by streamline length. These analyses confirmed the results of the main analysis. A detailed description can be found in the Supplementary Materials.

### Graph Theory

Graph theory was employed to investigate differences in network architecture between the *ZDHHC*9 and control group. To this end, graph metrics were calculated in the python implementation of the Brain Connectivity Toolbox https://sites.google.com/site/bctnet/. Weighted undirected networks were used for all analyses. The weight represented the FA value in the structural connectome. A detailed description of commonly used graph theory metrics can be found elsewhere (Bullmore and Sporns 2009; Rubinov and Sporns 2010).

### Allen Brain Atlas Data

Gene expression data were obtained from the Allen Brain Atlas Human Brain public database (http://human.brain-map.org). Gene expression data were based on microarray analysis of postmortem tissue samples from 6 human donors between 18 and 68 years with no known history of neuropsychiatric or neurological conditions (see online documentation). MRIs and transformations from individual donors MR space to MNI coordinates were also obtained from the Allen Brain Atlas website. For the current investigation, expression values were averaged across donors and mapped onto areas of the Desikan-Killiany parcellation of the MNI brain as described by French and Paus (2015). The current investigation focussed on the expression of *ZDHHC9*. In order to investigate the specificity of the link of *ZDHHC9* expression and structural connectome organization, we compared *ZDHHC9* to a number of other genes: First, *GAPDH* was added as a control gene that is not associated with any known neurological or cognitive phenotype (Nicholls et al. 2012). We then assessed genes that are associated with a similar mutation phenotype. For overlap with language deficits, *FOXP2* was included (Vargha-Khadem et al. 2005). *FMR1* was selected as an XLID gene (Bourgeois et al. 2009). *GRIN2A* was included for the association with Rolandic Epilepsy (McTague et al. 2016).

### Statistical Analysis

#### Comparison of Participant Groups

Participants in the *ZDHHC*9 and control group were matched on age (±2 years). Therefore, statistical comparisons were based on paired sample tests. Due to the rarity of single-gene disorders, the size of the sample was limited. Some controversy exists regarding optimal statistical procedures in small samples. Paired *t*-test comparisons are both robust to some violation of the normality assumption and to small sample sizes (Campbell et al. 1995; Bridge and Sawilowsky 1999; Fritz et al. 2012). In all cases, we also tested for any deviation from the normality assumption, using the Shapiro–Wilk test, which provides the best sensitivity (Razali and Wah 2011). Bonferroni correction was also applied to correct for multiple comparisons. For topographical analysis, false discovery rate (FDR) correction using the Benjamini–Hochberg method was applied. This maximizes power in the presence of a very large number of comparisons.

#### Regional Variation in Graph Measures and Association With Gene Expression and Group-Average Graph Metrics

Differences in node-level graph metrics were compared between groups. Deviations from the normality assumption were very rare, being present for only 3–5% of regions (node degree: 3.53, node strength: 5.88, clustering coefficient: 3.53, efficiency: 3.52). For this reason, we retained the paired-sample *t*-tests as our primary means of comparison—the statistical sensitivity of this method is superior to the nonparametric alternatives—but we disregarded those few instances where the normality assumptions were violated.

The linear association between gene expression and group-average graph metrics was investigated with linear regression models. Separate simple regression models were fitted with the graph metric as the outcome and gene expression and an intercept term as the predictor (model: Y_GraphMetric_ = β_GeneExpression_X_GeneExpression_ + β_Intercept_). Bonferroni correction was used to correct for multiple comparisons arising from the number of groups (*ZDHHC9*, control), the number of genes, and the number of graph metrics entered into the analysis.

## Results

**Figure 2:**
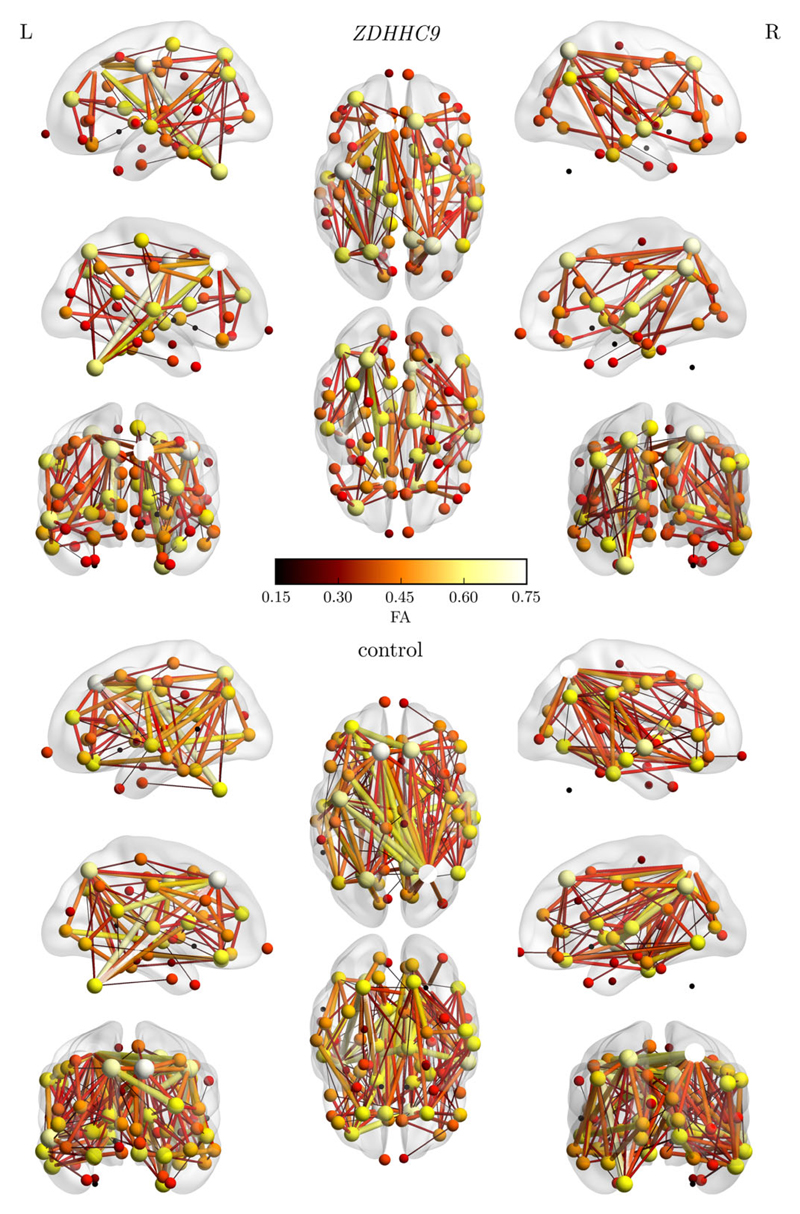
Illustration of the FA-weighted structural connectome in the *ZDHHC*9 and control group. The connection matrix was thresholded at a high cut-off at FA > 0.15 for illustration purposes.

The following section describes the results of the structural connectome comparison between the *ZDHHC9* group and controls. The analysis first focused on regional differences in edge weight between the groups as a basic property of the network. Next, graph theory was employed to characterize connectivity principles of the networks. Last, the relationship between regional variation of *ZDHHC9* expression and these connectivity properties was investigated. Illustrations of the topography of the structural network are presented in Figure 2. These illustrations were thresholded at a high cut-off to make the figure more readable. Unthresholded adjacency matrices of the group average networks can be found in the Supplementary Materials.

### Reduced Regional Edge Weight in the ZDHHC9

**Figure 3:**
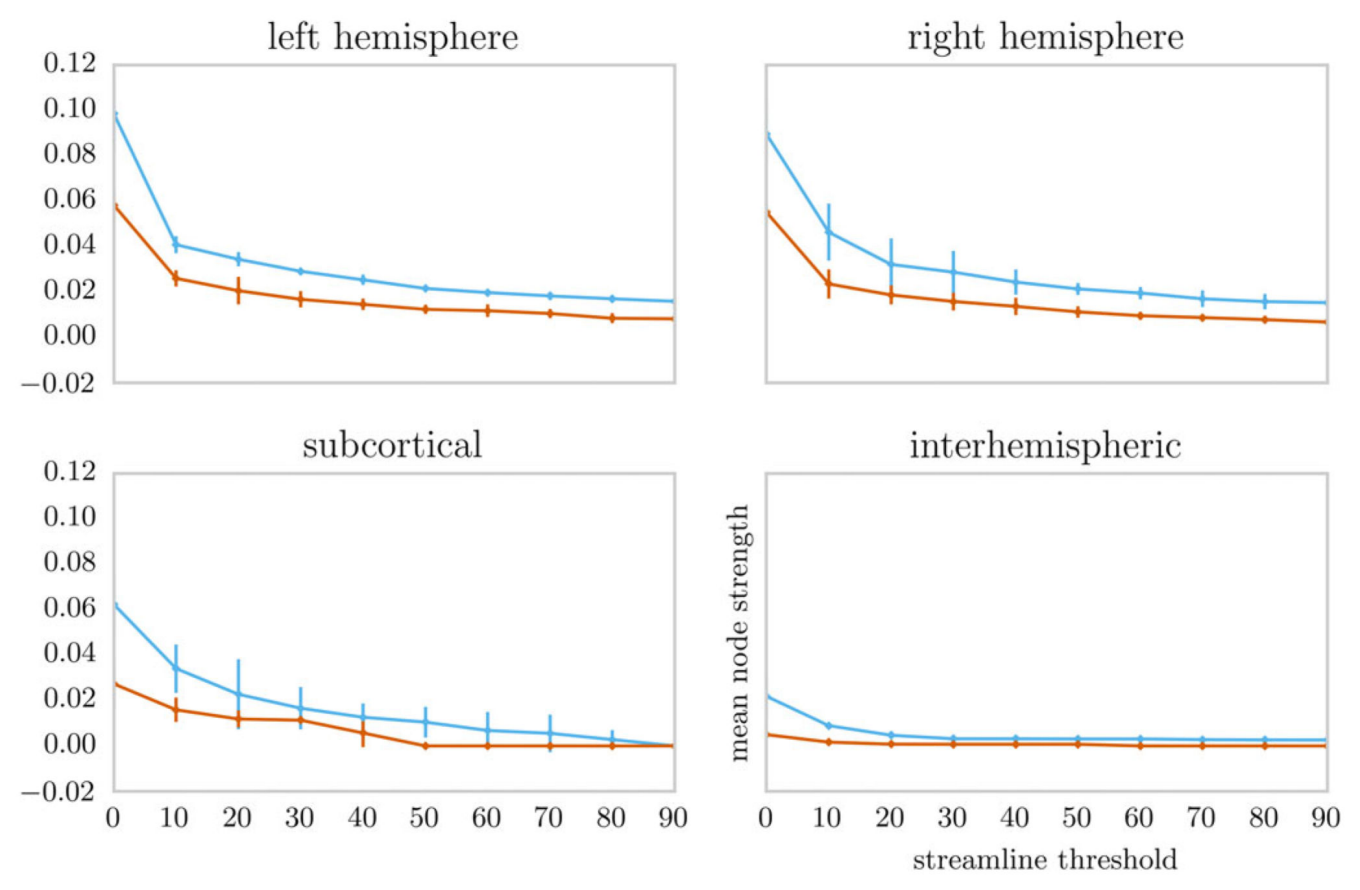
Comparison of node strength between the *ZDHHC*9 and control group for left and right hemisphere connections, subcortical–cortical connections, and interhemisphere connections. The line indicates the median in each group. The error bars indicate the bootstrapped 95% confidence interval around the median. The area under the curve across thresholds was used for statistical comparison between the groups to avoid the potential biasing effects of an arbitrarily selected threshold (Wijk et al. 2010).

Comparison of edge weights by region indicated a significantly lower edge weight in the *ZDHHC9* group for subcortical-cortical, left hemisphere cortical, right hemisphere cortical, and interhemispheric connections (Table 1 and Fig. 3).

### Reductions in Global Graph Metrics in the ZDHHC9 Group

**Figure 4:**
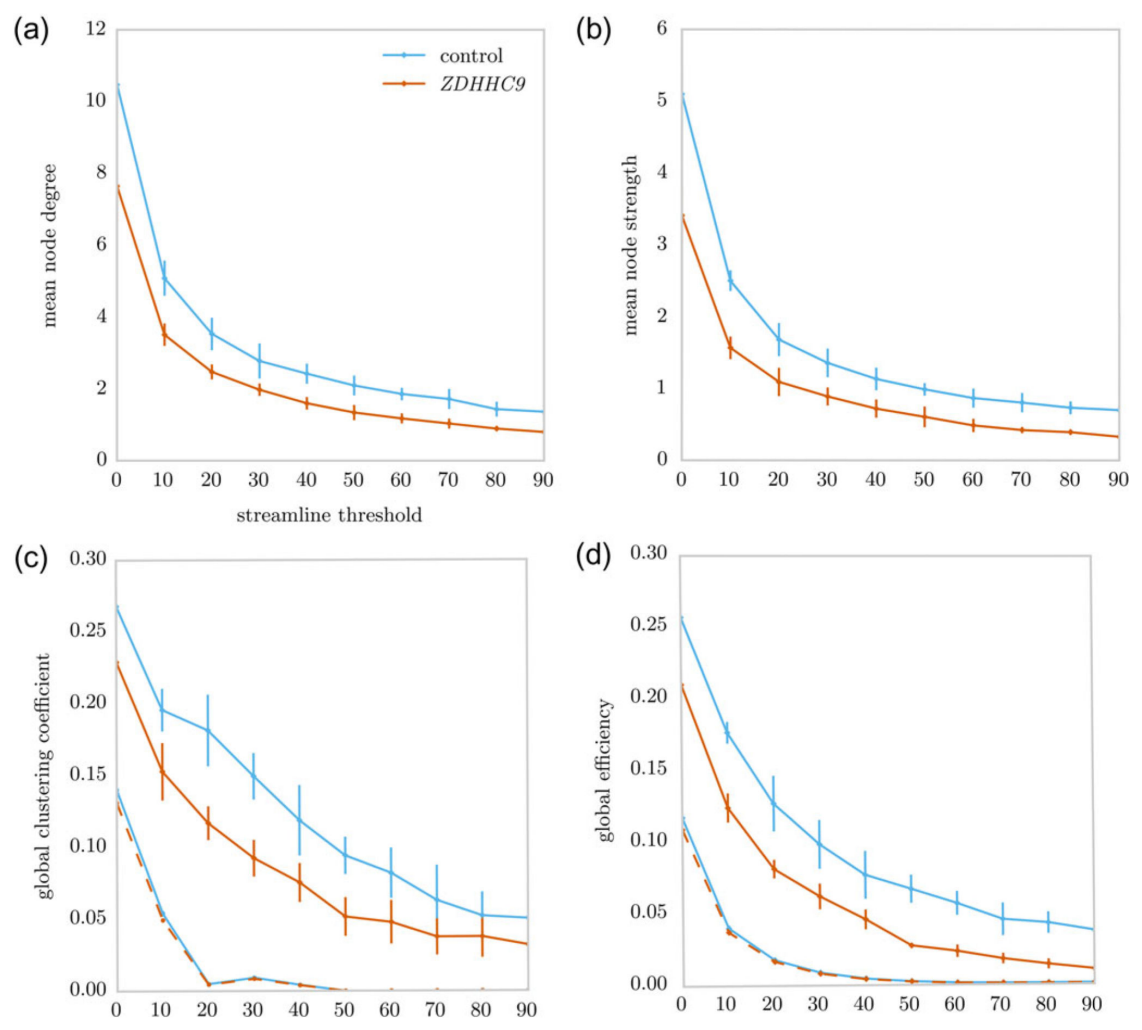
Comparison of global graph metrics between the *ZDHHC9* (orange) and control group (blue) across a range of streamline thresholds for (*a*) mean node degree, (*b*) mean node strength, (*c*) clustering coefficient, and (*d*) global efficiency. The line indicates the median value for each group. The error bars indicate the bootstrapped 95% confidence interval around the median. Panels (*c* and *d*) solid lines show the result for the native networks and dashed lines show results for networks after group consensus thresholding.

Statistical comparison indicated significant differences in mean node degree and mean node strength. Global clustering coefficient and global efficiency are significantly influenced by node degree. In order to adjust for differences in network density, group-level consensus thresholding was applied, such that we only retained connections that were found in each participant (de Reus and van den Heuvel 2013; Fornito et al. 2016). Analysis of global clustering coefficient and global efficiency in the consensus-thresholded networks indicated a significant reduction in both metrics in the *ZDHHC9* group (Table 2 and Fig. 4).

### Regional Reductions in Graph Metrics in the ZDHHC9 Group

**Figure 5:**
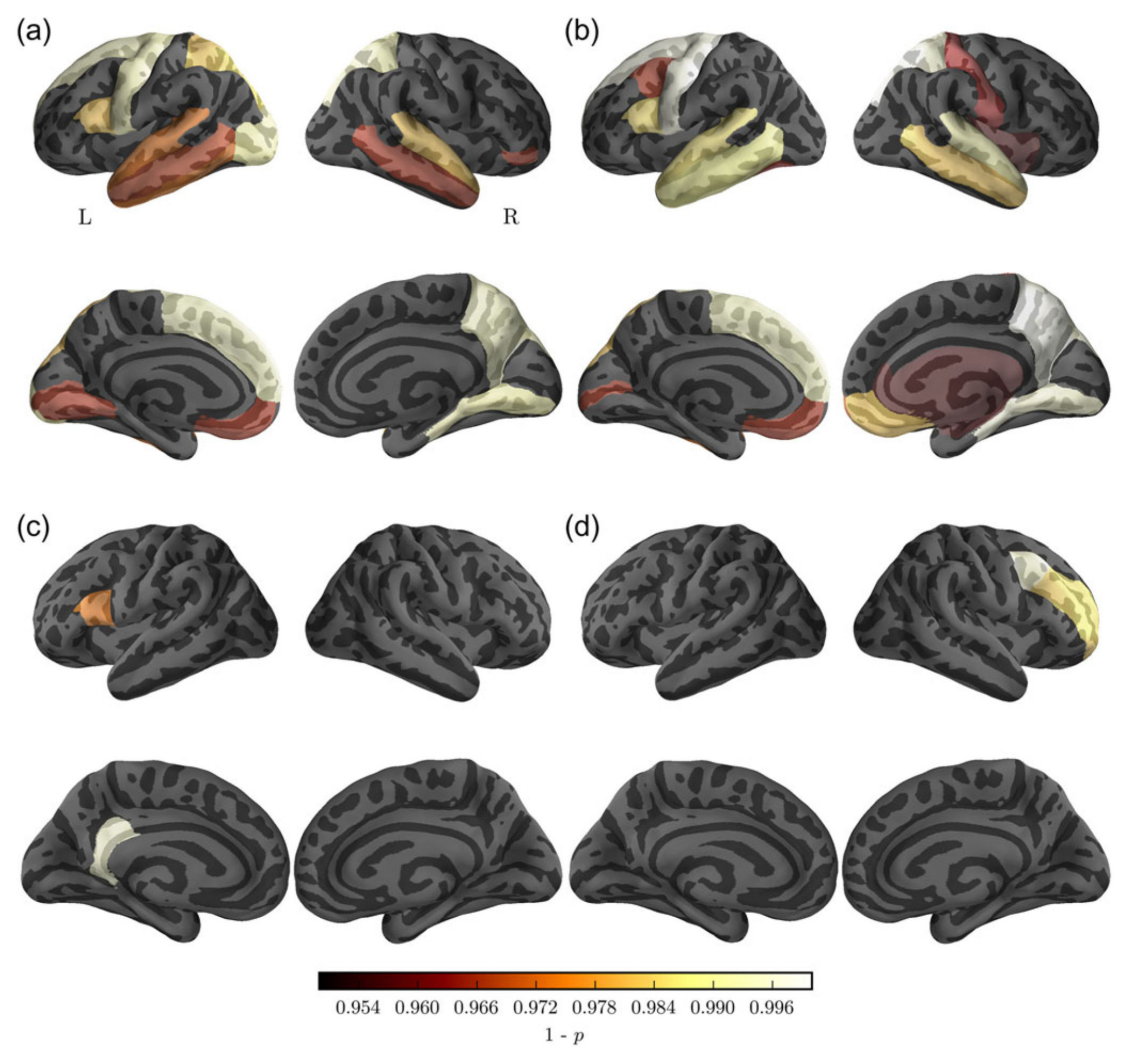
Comparison between the *ZDHHC*9 and control group in node measures of (*a*) node degree, (*b*) node strength, (*c*) clustering coefficient, and (*d*) local efficiency. The maps show *P*-values of paired-sample *t*-tests corrected for multiple comparison using false discovery rate (FDR).

Regional comparison of node degree and node strength indicated reduction in the *ZDHHC9* group for the brain stem, caudate, and putamen (Table 3a and b). Cortical differences were found in areas of the left and right temporal lobe, parietal lobe, and frontal lobe (Fig. 5*a* and *b*). Reductions in the local clustering coefficient in the *ZDHCC9* group were found for the left inferior frontal gyrus, right isthmus, and cingulate cortex (Table 3c and Fig. 5*c*). Local efficiency was found to be reduced around the right superior frontal cortex (Table 3d and Fig. 5*d*). There were no significant increases for any region or measure in the *ZDHHC9* group compared with controls.

### ZDHHC9 Expression and Structural Connectome Properties

**Figure 6:**
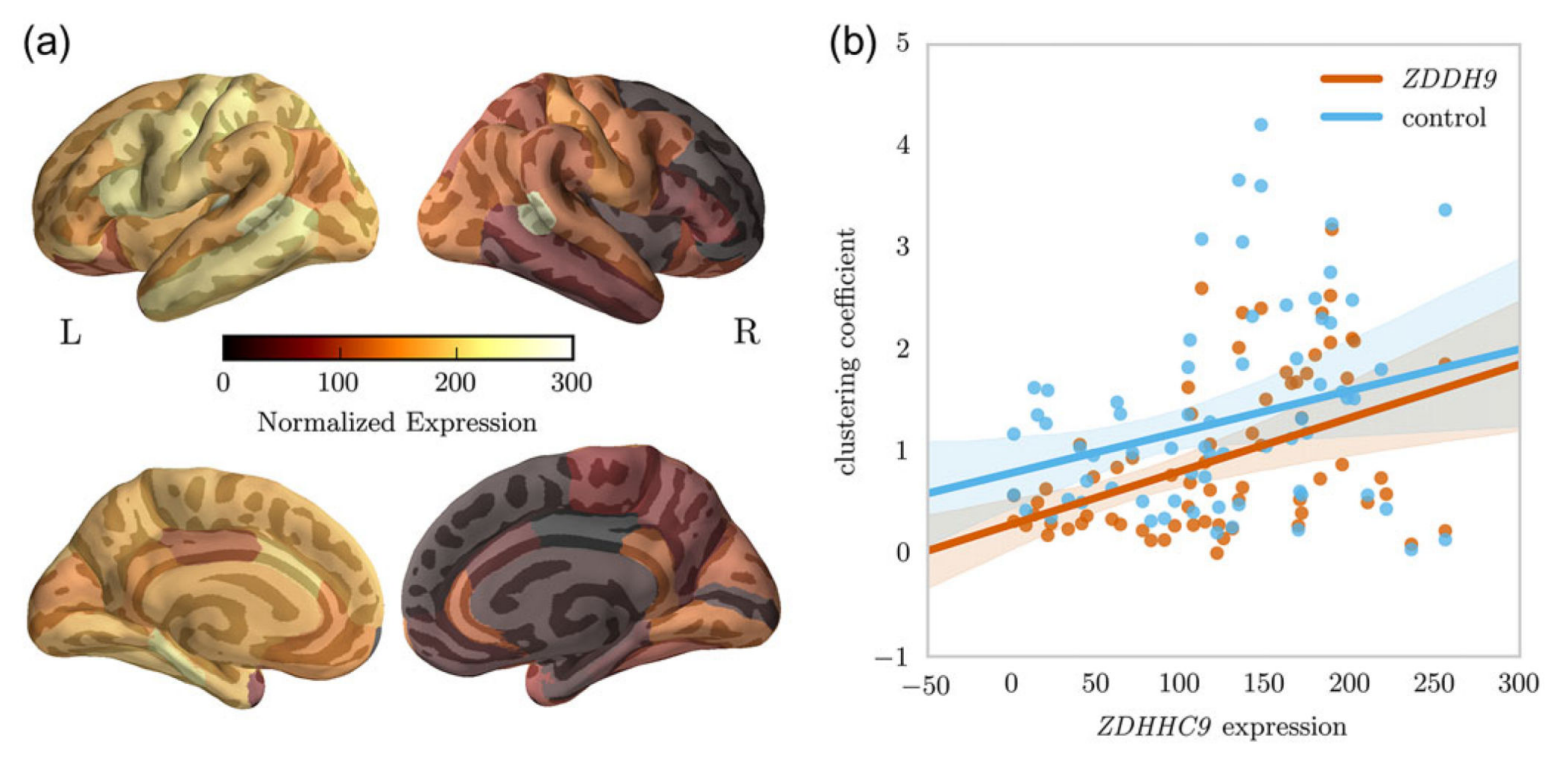
(*a*) Normalized expression of *ZDHHC9* across the cortex (*b*) Relationship between average node clustering coefficient in the *ZDHHC*9 and control group and normalized regional expression of *ZDHHC9*. Regression analysis indicated a significant positive relationship between clustering coefficient and *ZDHHC9* expression in the *ZDHHC9* group (Bonferroni-corrected: *P* = 0.003), but not the control group (Bonferroni-corrected, *P* = 0.444)

Normalized gene expression obtained from the Allen Brain Institute Human Brain database indicated higher expression of *ZDHHC9* in the left compared with the right hemisphere (see Fig. 6). Local maxima were found in the left postcentral gyrus, inferior frontal cortex, anterior cingulate cortex, inferior parietal lobule, and right lingual gyrus. Low expression was observed in the right posterior and isthmuscingulate cortex, and left superior temporal gyrus.

Next, the relationship between node-level graph metrics and gene expression in each region was investigated. The analysis indicated a significant positive association between node-level clustering coefficient and *ZDHHC9* expression in the *ZDHHC9* [*F*(1,66) = 15.62, *R*2 = 0.191, *β* = 0.0052, *P* < 0.001, corrected-*P* = 0.008, see Fig. 6], but not in the control group [*F*(1,66) = 5.486, *R*2 = 0.077, *β* = 0.002, *P* = 0.022, corrected-*P* = 0.888]. No significant association between graph measures and expression of other genes were found. Cook’s distance indicated the presence of 3 influential data points, that is, the left (*c* = 0.14) and right banks of the superior temporal sulcus (*c* = 0.18), and the left caudal anterior cingulate (*c* = 0.11). However, the association between *ZDHHC9* expression and clustering coefficient in the *ZDHHC9* group was also present when these regions were removed [*F*(1,63) = 23.68, *R*2 = 0.273, *β* = 0.006, *P* < 0.001, corrected-*P* = 0.001].

## Discussion

Loss of function mutations in *ZDHHC9* result in pervasive differences in white matter volumes and integrity (Bathelt et al. 2016), alongside a cognitive profile that includes profound expressive language deficits (Baker et al. 2015). Topographical analysis of clustering coefficient and local efficiency indicated differences in nodes of the frontal, left parietal, and right temporal lobe. These results suggest that these nodes are less integrated with the rest of the network in the *ZDHHC9* group. These regionally specific effects may provide a basis for the cognitive profile that these individuals show. Reduced connectivity between nodes of a network involving frontal and temporoparietal nodes is consistent with the previously described language deficits in this group (Baker et al. 2015). Deficits in inhibitory control (Baker et al. 2015) may arise from the reduced integration of nodes of the anterior cingulate and prefrontal cortex.

Furthermore, the regional variation in clustering coefficient within the *ZDHHC9* group is predicted by regional expression levels of *ZDHHC9*. The higher the expression patterns of *ZDHHC9*, the higher the clustering coefficient in this group. For example, expression of *ZDHHC9* is highest in left temporoparietal regions and frontal regions. The largest reduction in regional comparison of clustering coefficient in the *ZDHHC9* group were also found in a frontal region. This convergent finding supports the suggestion that *ZDHHC9* may play a critical role in shaping long-range white matter connectivity of these regions.

The influence of *ZDHHC9* mutation on structural brain organization shows both similarities and differences when compared with other groups with a similar phenotype or genetic mechanisms. Like *ZDHHC9* mutation (Raymond et al. 2007), Fragile-X syndrome (FXS) is a cause of XLID. Leow et al. (2014) investigated local and global properties of the white matter connectome in FXS. FXS is caused by CGG trinucleotide repeats in the Fragile-X mental retardiation 1 (*FMR1*) gene on the X chromosome (Belmonte and Bourgeron 2006). Leow and colleagues reported an association between the number of trinucleotide repeats in the *FMR1* gene and global network efficiency in male premutation carriers as well as local differences in efficiency and clustering coefficient in left temporal nodes (also see Bruno et al. 2016). Our results for *ZDHHC9* also indicated a reduction in global efficiency of the structural network similar to that reported for FXS, suggesting that this observation relates nonspecifically to low IQ. However, topographical analysis of clustering coefficient and local efficiency indicated reductions in the frontal lobe in the *ZDHHC9* group, whereas reductions in temporal areas were statistically indistinguishable from the control group. In other words, mutations in *ZDHHC*9 and *FXS* show a convergent reduction in global network efficiency, but different local patterns of efficiency and clustering coefficient that distinguish the groups.

Rolandic epilepsy is another relevant neurodevelopmental condition for comparison due to the overlapping phenotype of expressive language deficits and epilepsy with centro-temporal spikes that were also observed in the carriers of *ZDHHC9* mutation (Baker et al. 2015). A study by Besseling and colleagues identified a reduction in structural white matter connectivity of the Perisylvian system, including the left inferior frontal, supramarginal, and postcentral gyrus (Besseling et al. 2013b). Studies of functional connectivity indicated reduced integration of these areas and delayed convergence of structural and functional connectivity in RE (Besseling et al. 2013a, 2013b; Besseling et al. 2014). Further, graph theoretical analysis of the functional connectome indicated reduced clustering coefficient and local efficiency in areas of the parietal and frontal lobe in RE similar to the findings of structural connectivity differences in the current study (Xia et al. 2013). In summary, studies of functional and structural connectivity in a neurodevelopmental condition of mixed etiology with a similar phenotype to *ZDHHC9* mutation showed reduced connectivity in areas of the parietal and frontal lobe akin to the structural connectivity changes observed in the current investigation. We are not aware of another connectome analysis of a developmental language disorder (either of known or unknown origin) against which to compare the results of our study.

The findings of the current investigation are associated with some limitations. In addition to general limitation of diffusion-weighted imaging, such as low signal-to-noise ratio, issues regarding the fit of the diffusion model, presence of crossing fibers (Jones et al. 2013), there are specific limitations related to structural connectome approaches (Fornito et al. 2013). A multitude of methods for structural connectome analysis of diffusion-weighted MR data have been reported in the literature (Qi et al. 2015) and there is currently no consensus on best practices or published rigorous comparisons across different methods (Zalesky et al. 2010; Qi et al. 2015). One major issue is the presence of false positive and false negative connections associated with the tractography algorithm (Garrison et al. 2015; Zalesky et al. 2016). In ew of this ongoing debate within the field, we conducted various control analyses based on deterministic tracking along the maximum direction of an alternative diffusion model shown in the. These are included in the Supplementary Materials section, and show similar results as reported in the main analysis.

The connection weights in the current analysis were based on the average FA along the entire tract, which is likely to minimize some of the problems associated with streamline measures. However, this measure is also less sensitive to localized effects and may be influenced by crossing fibers. To address this, control analyses using a metric that is able to incorporate crossing fibers, that is, Generalized FA (Cohen-Adad et al. 2008), were conducted. The results of these converged with the findings of the main analysis, and are included in the Supplementary Materials.

Another limitation concerns the node definitions. The current study used a relatively coarse anatomical parcellation of the cortex and subcortical areas, because this enabled us to explore relationships with gene expression, which was only available in this parcellation scheme. Given the pervasive differences in diffusion tensor metrics at voxel resolution (Bathelt et al. 2016), we think that is unlikely that a more detailed parcellation would provide much additional information.

Further, the possible sample size of studies of this kind is inherently limited, because of the rarity of single gene mutations. Therefore, the current findings are based on a small sample, which increases the chance of false positive findings and may exaggerate effect sizes (Button, et al. 2013). However, investigations of homogeneous etiology groups as presented in the current work provide unique insight into the effect of single gene disorders that is not afforded in large heterogeneous samples of behaviorally defined groups. In a future study with larger number of participants and more detailed clinical and behavioral evaluations, it may be possible to correlate variation in neuroanatomical differences within the *ZDHHC9* group with specific outcomes.

## Conclusion

The current investigation aimed to elucidate the association between a neurodevelopmental disorder of known monogenic origin and white matter organization. Mutations in the *ZDHHC9* gene were associated with reductions in connection weight that resulted in altered network properties, including reduction in mean clustering coefficient and global efficiency. Topological analysis of these differences indicated that reductions in edge weight in the *ZDHHC9* group were most pronounced for frontal and temporo-parietal nodes. Furthermore, comparison of graph theory metrics with *ZDHHC9* expression data obtained from the Allen Brain Human Brain repository indicated that higher expression of *ZDHHC9* related to higher local clustering. The results of the study suggest that mutations in the palmitoylation gene *ZDHHC9* impact on large-scale white matter organization. The organization of white matter networks may represent an important intermediate phenotype to understand the effect of genetic mutations on cognitive development.

## Supplementary Material

Supplementary material are available at *Cerebral Cortex* online.

Supplementary Figure 1

## Figures and Tables

**Table 1:** Comparison of edge weight for subcortical-cortical (subcort.), left-hemispheric (lh), right hemispheric (rh), and interhemispheric (interhem.) connections in the *ZDHHC*9 and control group

	*ZDHHC9*				Control					*P*	Corr.-*P*
			
	Mean	SE	Shapiro-*W*	Shapiro-*P*	Mean	SE	Shapiro-*W*	Shapiro-*P*	*t*(6)		
Subcort.	0.08	0.011	0.97	0.92	0.18	0.018	0.93	0.54	5.32	0.002	0.007
lh	0.19	0.012	0.98	0.95	0.33	0.012	0.82	0.07	11.68	<0.001	<0.001
rh	0.16	0.010	0.82	0.06	0.30	0.022	0.90	0.36	7.02	<0.001	0.002
Interhem.	0.01	0.004	0.84	0.11	0.06	0.005	0.91	0.38	8.76	<0.001	<0.001

**Table 2:** Comparison of graph metric between the *ZDHHC*9 and control group. Statistical comparison based on the area under the curve over different streamline count thresholds indicated lower node degree and node strength in the *ZDHHC9* group. Comparison in group-consensus-thresholded networks also indicated a lower global clustering coefficient and lower global efficiency in the *ZDHHC9* group

	*ZDHHC9*				Control				*t*(6)	*P*	Corr.-*P*
				
	Mean	SE	Shapiro-*W*	Shapiro-*P*	Mean	SE	Shapiro-*W*	Shapiro-*P*			
Degree	22.23	0.921	0.96	0.85	33.18	1.056	0.9	0.34	10.14	<0.001	<0.001
Strength	9.57	0.487	0.97	0.86	16.34	0.645	0.9	0.31	13.08	<0.001	<0.001
Clust. coef.	0.20	0.003	0.90	0.34	0.21	0.003	0.95	0.75	5.91	0.001	0.004
Efficiency	0.18	0.003	0.85	0.12	0.20	0.002	0.90	0.32	5.81	0.001	0.005

**Table 3:** Regional comparison of (a) node degree, (b) node strength, and (c) clustering coefficient, and (d) local efficiency (node eccentricity). Comparisons were corrected for multiple comparison using false discovery rate correction (Benjamini–Hochberg)

	*ZDHHC9*				Control				*t*(6)	*P*	Corr.-*P*
				
	Mean	SE	Shapiro-*W*	Shapiro-*P*	Mean	SE	Shapiro-*W*	Shapiro-*P*			
(a) Node degree										
Left-caudate	26.71	3.220	0.87	0.193	44.57	2.861	0.88	0.241	4.14	0.006	0.031
Right-caudate	19.29	2.661	0.83	0.088	33.86	2.586	0.92	0.472	4.85	0.003	0.020
Right-putamen	17.71	4.927	0.91	0.392	36.86	5.938	0.92	0.493	4.34	0.005	0.028
lh-inferiortemporal	14.29	2.466	0.90	0.336	28.57	1.850	0.86	0.156	4.33	0.005	0.028
lh-medialorbitofrontal	23.43	1.412	0.96	0.800	38.00	2.837	0.91	0.405	3.83	0.009	0.035
lh-middletemporal	10.71	1.248	0.86	0.157	18.71	1.267	0.87	0.195	4.00	0.007	0.034
lh-parsopercularis	11.43	2.369	0.92	0.499	22.14	1.752	0.90	0.331	5.16	0.002	0.018
lh-pericalcarine	22.00	2.000	0.82	0.070	36.14	2.198	0.97	0.913	3.87	0.008	0.035
lh-precentral	60.86	4.803	0.95	0.723	107.43	4.064	0.83	0.078	7.36	0.000	0.008
lh-superiorfrontal	76.29	5.402	0.86	0.140	135.71	6.643	0.91	0.425	8.55	0.000	0.006
lh-superiorparietal	50.14	5.049	0.83	0.079	85.29	5.126	0.83	0.088	5.15	0.002	0.018
lh-superiortemporal	50.57	2.125	0.95	0.728	65.29	2.222	0.93	0.569	4.39	0.005	0.028
rh-lingual	13.43	2.742	0.96	0.819	34.86	3.453	0.91	0.366	6.51	0.001	0.009
rh-middletemporal	22.00	2.370	0.93	0.525	39.14	4.636	0.96	0.800	3.75	0.010	0.035
rh-parahippocampal	7.43	1.232	0.88	0.249	20.43	1.395	0.87	0.205	7.17	0.000	0.008
rh-parsorbitalis	7.29	0.837	0.93	0.555	13.71	1.822	0.83	0.089	3.72	0.010	0.035
rh-precuneus	54.00	3.748	0.95	0.735	98.86	6.296	0.91	0.423	9.40	0.000	0.006
rh-superiorparietal	62.71	5.317	0.97	0.923	125.57	5.559	0.86	0.151	6.93	0.000	0.008
rh-superiortemporal	40.57	4.418	0.82	0.063	71.29	2.917	0.96	0.844	5.31	0.002	0.018
(b) Node strength										
Brain-stem	117.17	5.576	0.94	0.594	144.22	5.829	0.91	0.401	4.37	0.005	0.018
Right-caudate	9.20	1.258	0.89	0.262	17.23	1.552	0.97	0.920	4.43	0.004	0.018
Right-putamen	7.09	2.028	0.90	0.336	16.58	3.006	0.95	0.717	4.21	0.006	0.020
lh-caudalmiddlefrontal	9.15	0.583	0.97	0.910	13.81	1.210	0.99	0.989	3.48	0.013	0.035
lh-fusiform	14.26	2.449	0.92	0.495	23.41	1.717	0.94	0.626	3.39	0.015	0.037
lh-inferiortemporal	6.38	1.339	0.90	0.341	14.66	0.787	0.97	0.925	5.38	0.002	0.011
lh-isthmuscingulate	5.23	1.205	0.96	0.855	14.28	1.174	0.92	0.502	4.84	0.003	0.014
lh-lingual	8.22	0.383	0.87	0.175	13.08	0.790	0.91	0.368	6.28	0.001	0.008
lh-medialorbitofrontal	8.76	0.465	0.91	0.406	17.92	2.140	0.86	0.139	3.68	0.010	0.031
lh-middletemporal	4.72	0.576	0.81	0.051	9.90	0.613	0.85	0.121	5.40	0.002	0.011
lh-parsopercularis	5.17	1.095	0.94	0.632	10.51	1.149	0.92	0.482	4.69	0.003	0.015
lh-pericalcarine	10.18	1.085	0.82	0.058	20.07	1.291	0.94	0.618	4.99	0.002	0.014
lh-precentral	30.02	2.804	0.93	0.515	57.94	2.438	0.86	0.138	9.04	0.000	0.003
lh-rostralanteriorcingulate	6.98	1.126	0.93	0.531	15.70	1.952	0.92	0.466	4.13	0.006	0.021
lh-superiorfrontal	36.07	3.302	0.93	0.576	70.83	3.858	0.98	0.939	9.98	0.000	0.002
lh-superiortemporal	22.68	0.827	0.92	0.489	33.89	1.677	0.87	0.195	4.87	0.003	0.014
rh-lingual	5.38	1.233	0.97	0.932	18.49	1.984	0.92	0.499	6.99	0.000	0.005
rh-medialorbitofrontal	5.76	0.739	0.93	0.561	11.10	0.872	0.99	0.990	4.39	0.005	0.018
rh-middletemporal	8.15	0.933	0.91	0.364	19.10	2.706	0.98	0.943	4.39	0.005	0.018
rh-parahippocampal	3.27	0.613	0.84	0.100	10.74	0.728	0.89	0.288	8.32	0.000	0.003
rh-postcentral	3.99	1.483	0.86	0.168	14.66	2.370	0.93	0.528	3.36	0.015	0.037
rh-precuneus	26.26	2.302	0.94	0.637	52.14	3.591	0.96	0.807	12.42	0.000	0.001
rh-rostralanteriorcingulate	5.60	0.727	0.95	0.761	11.87	2.223	0.82	0.069	3.17	0.019	0.044
rh-superiorparietal	29.04	2.939	0.96	0.789	64.64	3.058	0.87	0.169	7.39	0.000	0.004
rh-superiortemporal	18.20	2.316	0.89	0.258	35.87	1.937	0.95	0.716	6.02	0.001	0.008
rh-frontalpole	2.40	0.683	0.81	0.054	4.45	0.565	0.85	0.111	3.74	0.010	0.031
(c) Clustering coefficient										
rh-insula	2.73	0.208	0.83	0.077	5.68	0.951	0.88	0.214	3.13	0.020	0.044
lh-isthmuscingulate	1.07	0.433	0.84	0.098	3.62	0.287	0.90	0.333	10.01	0.000	0.005
lh-parsopercularis	0.75	0.157	0.90	0.341	1.81	0.162	0.91	0.409	6.39	0.001	0.027
(d) Local efficiency										
rh-caudalmiddlefrontal	76.81	5.653	0.90	0.345	120.94	4.369	0.91	0.401	10.00	0.000	0.005
rh-rostralmiddlefrontal	83.09	4.541	0.92	0.504	124.14	6.102	0.95	0.725	6.56	0.001	0.017
