# Supplementary figures and images for: Global and Local Connectivity Differences Converge With Gene Expression in a Neurodevelopmental Disorder of Known Genetic Origin
Source: Cereb Cortex. Author manuscript; Available in PMC 2019 Jul 1. (PMC6600876; doi:10.1093/cercor/bhx027)

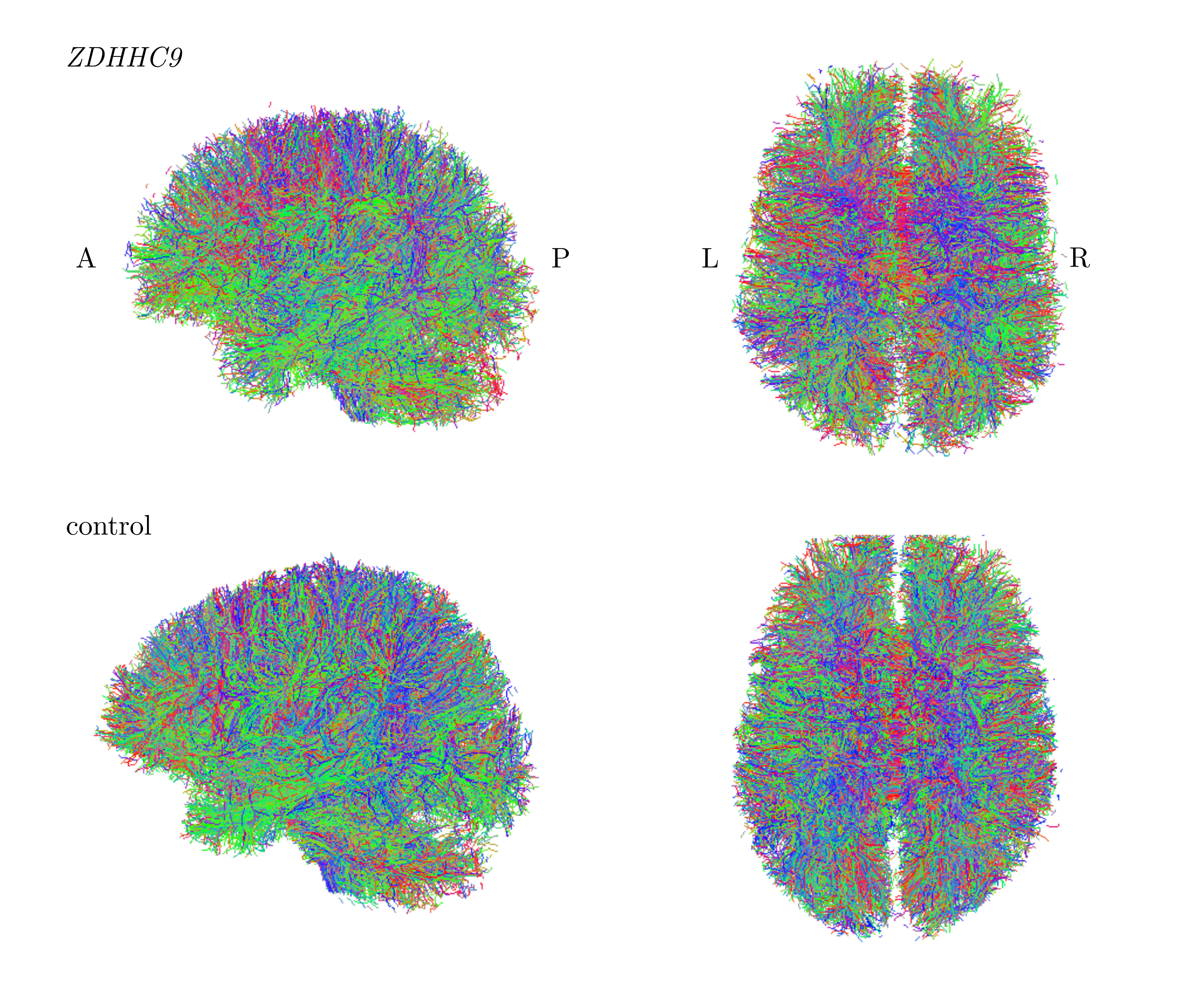

Supplement: Supplementary Figure 1 [file EMS83496-supplement-Supplementary_Figure_1.png]
